# OR10-005 - Treatment responses in TRAPS: Eurofever/ Eurotraps

**DOI:** 10.1186/1546-0096-11-S1-A188

**Published:** 2013-11-08

**Authors:** HJ Lachmann, K Minden, L Obici, A Naselli, A Insalaco, V Hentgen, R Merino, C Modesto, N Toplak, R Berendes, R Cimaz, A Jansson, A Martini, P Woo, I Touitou, M Gattorno

**Affiliations:** 1 for PRINTO, Eurotraps/Eurofever Projects, Genoa, Italy

## Introduction

TRAPS is a rare lifelong disease. Optimal treatment is not established but tends to rely either on corticosteroids with risks of known short and long term side effects or anti cytokine agents which are expensive, and both types of agents lead to higher risk of infection.

## Objectives

To analyze treatments used and their responses in patients with clinical TRAPS associated with a pathogenic sequence variants (PSV) in TNFRSF1A enrolled in the Eurofever/Eurotraps registry.

## Methods

The Eurofever Project (agreement n 2007332, EAHC) built a common web-based registry for all Autoinflammatory diseases in collaboration with the Eurotraps Project (FP7, HEALTH-F2-2008-200923).

## Results

In total there was treatment data on 113 patients with 45 different PSV of *TNFRSF1A*. Patients came from 14 countries and 94.5% were of European Caucasian ancestry. 16 patients had only received symptomatic treatment.

Of 48 patients given steroids only with attacks 20 (42%) reported complete success (CR) in terminating acute attacks but 38 (79%) were either converted to biologic therapy or had them added to improve disease control. Of 22 patients on maintenance steroids 6 (27%) reported complete attack prevention but 14 (64%) were converted to biologic therapy. 37 patients received etanercept (in the 19 where data was available for a median of 51 months). 9 patients had a CR and 26 a partial response (PR). 10 remain on etanercept. Of the 27 who discontinued etanercept inadequate disease response was sole or contributory reason for discontinuing etanercept in 21 and side effects in 9. 20 patients converted to anti IL-1 therapy. 38 patients received anakinra. 34 (89%) reported a CR and 4 a PR. 92% remain on anakinra with a median treatment duration date of 23 months (range 1- 89 months).

## Conclusion

This is the largest survey of treatment of TRAPS to date. The marked predominance of patients from Western Europe may be reflected in the high use of biologic agents which are not necessarily widely available. The most significant findings are that corticosteroids are effective in more than 40% of patients initially but almost 80% of patients have been converted to anti cytokine agents. Anakinra is completely effective in 89% of cases and continued as long-term treatment in 92%. Its use is associated with a 90% reduction in the requirement for corticosteroids to treat acute attacks. Etanercept is significantly less effective and is discontinued in almost 75% of cases. Although these data strongly support use of anti IL-1 agents to treat TRAPS follow up remains short and re-evaluation will be required.

## Competing interests

None declared.

